# Dr. I. V. C. Smith

**Published:** 1850-12

**Authors:** 


*Dr. I. V. C. Smith.*—The Boston Medical and Surgical Journal contains a series of letters from the Editor, Dr. Smith, who is making the tour of Europe. Dr. S. is awake to every thing interesting, and we anticipate the collection of his letters in a volume, which will be read with pleasure and profit by many who do not have access to his Journal.

